# Arsenic exposure and its implications in male fertility

**DOI:** 10.1590/1984-3143-AR2022-0119

**Published:** 2023-02-10

**Authors:** Mariana Machado-Neves

**Affiliations:** 1 Departamento de Biologia Geral Universidade Federal de Viçosa Viçosa MG Brasil Departamento de Biologia Geral, Universidade Federal de Viçosa, Viçosa, MG, Brasil

**Keywords:** arsenite, epididymis, male reproduction, oxidative stress, mitochondria

## Abstract

Arsenic exposure is a global health concern. This toxic metalloid is ubiquitous in the environment and contaminates food and drinking water. Once ingested, it undergoes a complex metabolic process within the body, which contributes to its accumulation and reactivity. Arsenic toxicity stems from the induction of oxidative stress, inhibition of thiol-containing proteins, and mimicry of inorganic phosphates. Arsenic poisoning is associated with the development of reproductive disorders. In males, arsenic causes a reduction in testicular weight and alterations in steroidogenesis and spermatogenesis. Moreover, it reduces the number and quality of spermatozoa harvested from the cauda epididymis. The mitochondria are targets of arsenic toxicity because of the production of free radicals and their high content of cysteine-rich proteins and fatty acids. Mitochondrial dysfunction may contribute to reproductive disorders because this organelle is crucial for controlling testicular and epididymal events related to sperm production and maturation. All of these alterations mediated by arsenic exposure contribute to the failure of male reproductive competence by reducing gamete viability. This review describes the potential mechanisms of arsenic toxicity, its detrimental effects on male reproductive organs, and consequences on sperm fertility.

## Introduction

Male fertility has deteriorated over the past few decades, with semen quality showing an evident downward trend (Miyaso et al., 2022). Overall, the decline in sperm quality involves the generation of high levels of oxidative stress in male reproductive organs driven by environmental and lifestyle factors (Aitken et al., 2016; Nowicka-Bauer and Nixon, 2020; Wu et al., 2020). Amongst environmental pollutants, arsenic, cadmium, lead, and mercury are hazardous to human and animal reproduction because of their toxic effects even at low levels, affecting the functionality of male reproductive organs and the ability of spermatozoa to fertilize oocytes and sustain initial embryonic development. These heavy metals do not exert known biological functions, accumulate within the organs, and persist in the food chain (Wirth and Mijal, 2010; Aitken et al., 2012; Hardneck et al., 2018). The intensity of their toxicity depends on route of exposure, dose, frequency, and chemical species, as well as age, gender, genetics, and nutritional status of exposed individuals (Tchounwou et al., 2012; Wu et al., 2016; Machado-Neves, 2022). Heavy metal poisoning in the livestock industry has a negative impact on animal fertility and productivity, with possible contamination of milk and meat, which represents a silent economic loss and serious food safety problem (Verma et al., 2018; Guvvala et al., 2020; Wrzecinska et al., 2021).

In contrast to other heavy metals, arsenic is a metalloid element exhibiting properties of metals and nonmetals, with toxicity potential related to its chemical form and metabolism within the body. Arsenic can be found in its organic and inorganic states with different valences (-3, 0, +3 [arsenite], and +5 [arsenate]), relying upon its binding with oxygen, chlorine, sulfur, carbon, or hydrogen (Kim and Kim, 2015). Inorganic arsenite and arsenate are available for poisoning by natural and anthropogenic activities. Accordingly, they contaminate groundwater, soil, dust, and air, promoting human and animal exposure by involuntary ingestion of drinking water and food (Wang et al., 2006; Abdul et al., 2015; Das et al., 2021). Once ingested, inorganic arsenicals undergo several metabolic steps, mediated by gut bacteria and host enzymes, resulting in organic arsenic (Hirano, 2020). The underlying mechanism by which arsenic impairs testis, epididymis, and sperm function has been extensively investigated. To date, it seems that toxic insults caused by arsenic are associated with mitochondrial dysfunction and free radical-mediated toxicity, involving the generation of oxidative stress, interaction with thiol-containing proteins, and competition with inorganic phosphates (Pi) (Hu et al., 2020; Machado-Neves, 2022).

Some documented effects of arsenic exposure on human reproduction include erectile dysfunction, prostate cancer, and testicular disorders (Sun et al., 2017; Hardneck et al., 2018). A recent meta-analytical study evaluated the consequences of toxic metal exposure on domestic ruminants, demonstrating the deleterious effects of arsenic on andrological parameters, sperm motility, and sperm viability (Ribeiro et al., 2022). These findings suggest that high arsenic content in male reproductive organs can lead to subfertility/infertility. This review describes the effects of arsenic on male reproductive organs and potential mechanisms of toxicity. Arsenic exposure may affect sperm parameters directly and indirectly, compromising gamete fecundity.

## Arsenic metabolism and toxicity

**Figure 1:**
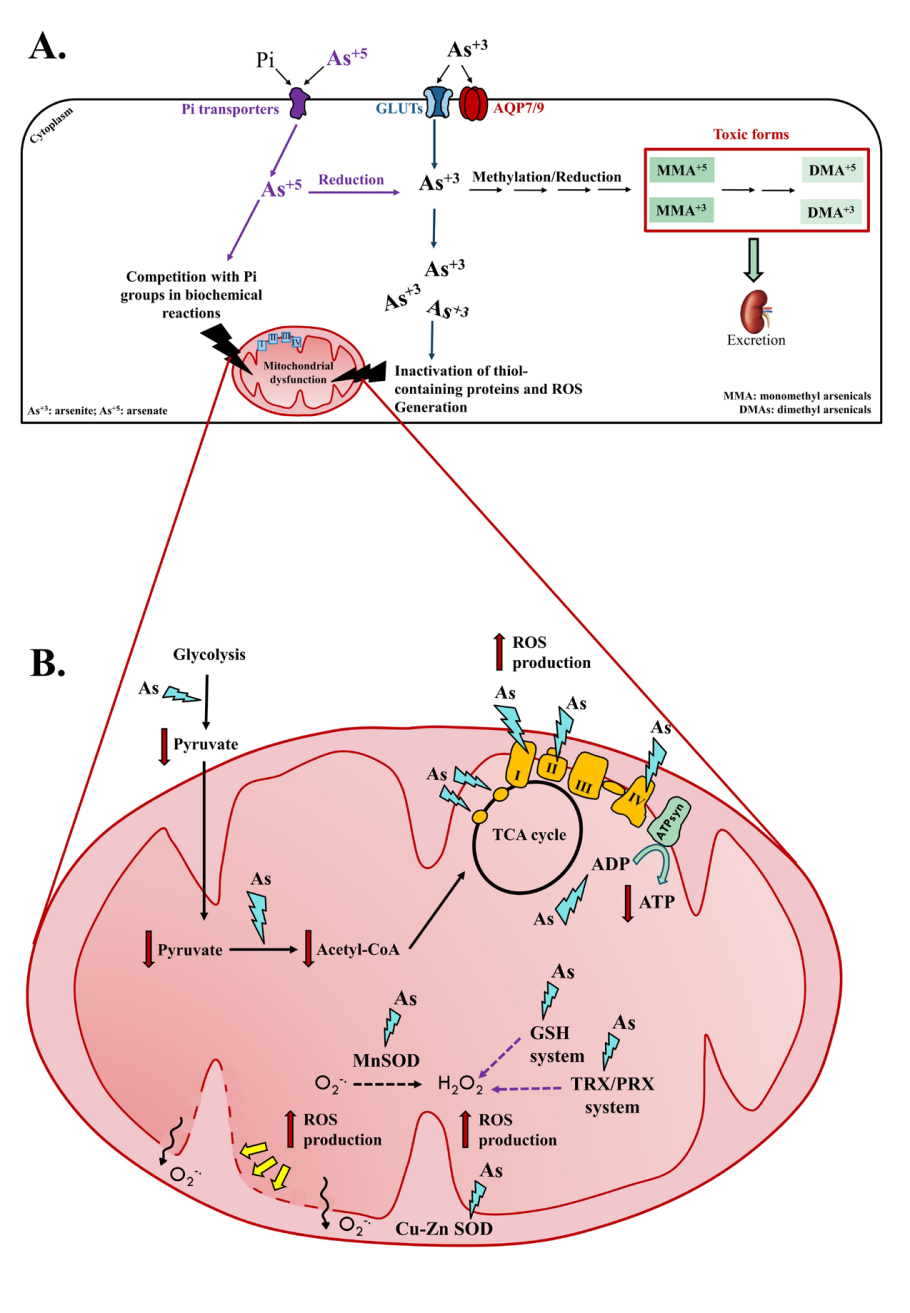
A) Arsenate (As^+5^) and arsenite (As^+3^) enter the cell through inorganic phosphate (Pi) transporters, glucose transporters (GLUTs), and aquaglyceroporins (AQP) 7 and 9. Once inside, As^+5^ is reduced in As^+3^ or can replace Pi groups in substrates and enzymes from biochemical reactions, such as glycolysis, oxidative phosphorylation, and ATP production. In turn, As^+3^ is metabolized through alternate methylation and reduction reactions to produce organic arsenic. This compound can also create an oxidized environment by ROS overproduction and bind to cysteine residues in protein/ enzymes with consequent misfolding and inactivation. B) Overall, arsenic (As) disturbs glycolysis and reduces pyruvate content. Meanwhile, this metalloid can inactivate pyruvate dehydrogenase, reducing the content of acetyl CoA. In addition to this, As inhibits the activity of succinate dehydrogenase and alpha-ketoglutarate dehydrogenase, blocking the activity of the tricarboxylic acid cycle (TCA cycle). Moreover, As binds to active sites from mitochondrial complexes disrupting oxidative phosphorylation and ATP production, as well as generating reactive oxygen species (ROS). Arsenic inhibits the activity of antioxidant enzymes, such as manganese and copper-zinc superoxide dismutase (MnSOD and Cu-Zn SOD, respectively), as well as enzymes from glutathione (GSH) system and thioredoxin (TRX) and peroxiredoxin (PRX) systems. The consequence of this mechanism is the overload of superoxide anion (O_2_^-.^) and hydrogen peroxide (H_2_O_2_) that, in turn, attack components of mitochondrial structure including the inner mitochondrial membrane, promoting the formation of mitochondrial permeability transition pores (yellow arrows). ATP: adenosine triphosphate; ADP: adenosine-5’-diphosphate; ATP syn: ATP synthase.

Arsenic compounds are metabolized to facilitate their elimination by the kidney, but at the same time, it contributes to arsenic accumulation and reactivity. Pentavalent arsenate crosses cell membranes through Pi transporters, whereas trivalent arsenite enters cells through aquaglyceroporins 7 and 9 and glucose transporters (Figure 1A). In the intracellular compartment, arsenate can replace Pi groups in substrates and enzymes from biochemical reactions (Figure 1A) and react with endogenous reductants, such as glutathione (GSH), which is converted into arsenite (Watanabe and Hirano, 2013). Arsenite, in turn, can conjugate with GSH for further methylation, bind to sulfhydryl (thiol)-containing proteins, and drive mitochondrial reactive oxygen species (ROS) generation (Figure 1A). Arsenic metabolism alters oxidative methylation and reduction reactions that modify oxidative states (+3 and +5) and methylation levels (monomethyl and dimethyl) of intermediate metabolites, forming toxic forms of organic arsenicals that enable their excretion through urine (Hirano, 2020; Thomas, 2021; Machado-Neves and Souza, 2022).

Arsenic toxicity mechanisms may disrupt cell metabolism by exacerbating the production of ROS and reactive nitrogen species (RNS), thereby inhibiting the antioxidant defense system and misfolding proteins/enzymes with subsequent inactivation. Accordingly, gene expression and cellular signaling pathways, including extracellular signal-regulated kinase, nuclear factor erythroid 2-related factor 2 (Nrf2)-antioxidant response element, and mitogen-activated protein kinase are altered (Valko et al., 2005; Rahaman et al., 2021). Oxidative stress induced by arsenic involves the excessive production of ROS/RNS (e.g., superoxide radical anion [O_2_^-^], hydrogen peroxide [H_2_O_2_], hydroxyl radicals, singlet oxygen, hydroperoxyl radical, peroxyl radical, and nitric oxide), thereby overloading the protective capacity of intrinsic antioxidant mechanisms to inhibit oxidation reactions, including non-enzymatic (e.g., alpha-tocopherol, ascorbic acid, retinol, molecules with thiol groups [GSH and lipoic acid], and transition-metal ions [Fe^+2^, Cu^+2^, Zn^+2^, and Mn^+2^]) and enzymatic scavengers, such as superoxide dismutase (SOD), catalase, glutathione peroxidase (GPX), glutathione reductase, peroxiredoxins (PRXs), and thioredoxin (TRX) (Moldogazieva et al., 2018; Aitken, 2020; Machado-Neves, 2022).

Under arsenic conditions, high levels of ROS lead to oxidation of proteins, lipids, and DNA, culminating in structural and functional damage. Additionally, ROS/RNS may interact with thiol-containing enzymes, thereby disturbing mitochondrial function, antioxidant activity, and hormone secretion. Lipids, in turn, suffer ROS attacks, causing membrane lesions that ultimately upregulate cell death-related proteins, including p53, Bax, LC3, and caspase-3, and downregulate survival proteins, such as mTOR, Akt, and Bcl-2. An oxidative microenvironment elicits gene instability, alterations in DNA methylation and repair, and DNA damage (base lesions and strand breaks), all of which contribute to arsenic genotoxicity and carcinogenicity (Valko et al., 2005; Rahaman et al., 2021). It is worth noting that ROS determine cell fate by participating in redox signals responsible for regulating and triggering several cellular events. Hence, redox reactions occur in a state of dynamic equilibrium, whereby the loss and gain of electrons are carefully balanced. For instance, ROS/RNS modify proteins in a reversible manner at the level of thiol-containing residues, cysteine, and methionine, thereby causing changes in protein structure and function, affecting localization and physical interactions, and undergoing further post-translational modifications, including phosphorylation (Jones, 2008; Filomeni et al., 2015; O’Flaherty and Matsushita-Fournier, 2017).

## Mitochondrion as a target organelle for arsenic toxicity

The primary damage caused by arsenic poisoning is associated with mitochondrial oxidative injury. The mitochondrion is a double-membrane organelle that plays a pivotal role in energy metabolism and is a major source of ROS generation through the electron transport chain. Mitochondria are susceptible to arsenic toxicity because of their high levels of thiol-containing enzymes from metabolic pathways and antioxidant defense systems. After exposure, this metalloid binds to the lipoic acid moiety and sulfhydryl groups of mitochondrial enzymes, including pyruvate dehydrogenase, succinate dehydrogenase, and alpha-ketoglutarate dehydrogenase, thereby reducing their affinity for substrates and coenzymes (Prakash et al., 2016). Inhibition of these enzymes affects the tricarboxylic acid cycle and subsequently affects ATP production. Another consequence is the depletion of mitochondrial NADH content, which in turn favors ROS production, culminating in oxidative stress (Machado-Neves and Souza, 2022). Arsenic also downregulates mitochondrial complexes I, II, and IV, thereby inhibiting energy-linked reduction of NAD, oxidative phosphorylation, and ATP synthesis (Figure 1B) (Jomova and Valko, 2011; Jomova et al., 2011; Hu et al., 2020; Machado-Neves and Souza, 2022).

Furthermore, arsenic created an oxidative environment by affecting the activity of antioxidant enzymes (Figure 1B). Mitochondria contain manganese SOD in the matrix and copper-zinc SOD in the intermembrane space, both of which are responsible for dismutating O_2_^-^ to H_2_O_2_. The latter, in turn, is eliminated by the GSH, TRX2, and PRX systems. The activity of these enzymes depends on either thiol or selenol active sites. Hence, the interaction between these sites and arsenics inhibits their activity, resulting in ROS accumulation (Figure 1B). Additionally, antioxidant enzymes can be indirectly affected by arsenic by binding to their cofactors. For instance, arsenic binds to GSH and TRX2, which are required for GPX and PRX activity. Likewise, this metalloid may affect GSH and TRX reductases, which are enzymes involved in recycling GSH and TRX2 and are therefore necessary for ROS elimination (Shen et al., 2013; Machado-Neves and Souza, 2022). Subsequently, the generation of oxidative stress promotes damage to mitochondrial membranes. The outer and inner mitochondrial membranes have a high content of polyunsaturated fatty acids (PUFAs) that are vulnerable to ROS attack. The burst of free radicals and H_2_O_2_ associated with GSH depletion causes peroxidation of membrane PUFAs, with the formation of mitochondrial permeability transition pores (Figure 1B). This permeabilization culminates in the loss of membrane potential. Ultimately, membrane damage impairs mitochondrial dynamics, owing to dysregulation of mitofusion and fission, which are essential for mitochondrial morphology, DNA stability, and respiratory capacity (Prakash et al., 2022).

## Arsenic disrupts male reproductive functions

Arsenic accumulation in male reproductive tissues is well documented and causes disturbances in the physiological processes involved in fertile spermatozoa production, such as steroidogenesis, spermatogenesis, and sperm maturation (Machado-Neves, 2022). The common alterations described in males exposed to arsenic include reduction in male organ weight, serum testosterone levels, sperm number, viability, and motility. Studies have also described alterations in the activity of antioxidant enzymes, levels of oxidative metabolites, and histomorphometry of the testicular and epididymal components (Souza et al., 2016b, a; Zubair et al., 2017; Souza et al., 2019).

The testis is involved in steroidogenesis, with Leydig cells producing testosterone. Arsenic induces gonadal dysfunction by decreasing testosterone biosynthesis, thereby inhibiting the action of testicular steroidogenic enzymes and impairing the transcriptional activity of androgen receptors. Leydig cell morphometry and functionality are disrupted after arsenic exposure through oxidative damage and the subsequent activation of apoptotic pathways. This metalloid also affects the regulation of pituitary gland function with a subsequent reduction in LH and FSH secretion (Kim and Kim, 2015; Zeng et al., 2018; Ommati et al., 2019; Aydin and Orta-Yilmaz, 2022; Machado-Neves, 2022).

Furthermore, the testes play a vital role in sperm production. Spermatogenesis is a complex and asynchronous process that involves mitotic proliferation of spermatogonia, meiotic divisions of spermatocytes, and differentiation of spermatids. Growing evidence suggests that the mitochondria play an active role in regulating stem cell fate decisions and spermatogenic lineage commitment via signal transduction, protein modification, and epigenetic modulation (Zhang et al., 2022). Inhibition of glycolysis by arsenic dysregulates spermatogonia stem cell self-renewal and regenerative capacity (Park and Pang, 2021). Impairment of cellular redox balance and mitochondrial damage during arsenic exposure may trigger autophagy in the spermatogenic lineage. Autophagy is the process of self-protection in cells by recycling the intracellular metabolites of damaged proteins and organelles, which are then degraded by the lysosomal pathway (Chen et al., 2020). Arsenic interferes with spermiogenesis in the haploid round spermatid stage by disorganizing the elongation of spermatids, thereby altering the expression of the DDX25 gene and affecting axoneme flagellum formation and acrosome biogenesis (Han et al., 2020a, b). This metalloid can also downregulate transmembrane proteins from the blood-testis barrier, leading to the loss of cell interaction and interruption of spermatogenesis (Araújo-Ramos et al., 2021). Histological alterations observed in the testes of exposed animals showed the presence of vacuoles at the base of the seminiferous epithelium, probably related to the response of Sertoli cells to arsenic insult and epithelium degeneration (Souza et al., 2016b; Medeiros et al., 2019; Machado-Neves, 2022), in addition to shrinkage of seminiferous tubules with a reduction in their diameter and germ cell population (Zubair et al., 2017; Couto-Santos et al., 2020).

The epididymis is also affected by arsenic intoxication. In contrast to the testis, the impact of arsenic exposure on epididymal biology has been poorly explored, with several questions that need to be addressed (Machado-Neves, 2022). In rodents, this organ is a single convoluted tubule divided into four regions (initial segment, caput, corpus, and cauda), thereby expressing its own and overlapping genes, proteins, and signal transduction pathways (Domeniconi et al., 2016). The epididymal duct is lined by pseudostratified epithelium composed of principal, basal, and mitochondria-rich clear and narrow cells. Together, these cell types establish a unique luminal environment for spermatozoa maturation and storage. Although this organ displays a battery of antioxidant enzymes that protect spermatozoa against oxidative damage, arsenic is capable of disturbing the balance between ROS levels and the antioxidant system, thereby causing structural and functional damage to the epididymal epithelium (Breton et al., 2019; Medeiros et al., 2019; Couto-Santos et al., 2020; Machado-Neves, 2022). Damage to the structure and function of principal cells, for example, may affect the production of several epididymosome proteins responsible for mature sperm structure and function and protect them from oxidative injuries. Lesions in the basal cells, in turn, may compromise the cell crosstalk between epithelial cells and the production of antioxidant enzymes that are important for maintaining low levels of ROS during epididymal maturation (Breton et al., 2019; O’Flaherty, 2019; Wu et al., 2020). The functionality of clear and narrow cells may be affected by arsenic intoxication through mitochondrial dysfunction. These cell types create an acidic luminal environment by regulating carbonic anhydrase activity, endocytotic activity, and proton secretion by V-ATPase in the epididymis (Breton et al., 2016; Park and Pang, 2021).

The detrimental effects observed in spermatozoa retrieved from the cauda epididymis of arsenic-exposed animals include reduction in sperm motility and viability, along with increased oxidative damage to the mitochondria, membranes, and DNA (Machado-Neves, 2022). Excessive ROS levels generate a hostile environment in the epididymis, which enhances the peroxidation of PUFAs from sperm membranes, thereby affecting sperm motility and viability. However, the affinity of arsenic to thiol groups elicits alterations in flagellar protein structure and mitochondrial function, thereby impairing sperm movements and ATP production, respectively (Guvvala et al., 2016; Lima et al., 2018). Recently, Couto-Santos et al. (2021) revealed that arsenic exposure may alter the epididymal sperm proteome, mainly the phosphoprotein profile, with consequent impairment of sperm fertility potential. The sensitivity of male gametes to ROS attack may be explained by the impossibility of spermatozoa to translate proteins, which is completely dependent on antioxidant protection by enzymes during spermatogenesis and epididymal maturation (O’Flaherty, 2019; Chianese and Pierantoni, 2021). Despite the scarcity of information regarding fertility indices from intoxicated animals, it is possible to state that disturbances on the microenvironment wherein spermatozoa are exposed to negatively influence the indices of male fertility potential and preimplantation and post-implantation losses (Lima et al., 2018; Couto-Santos et al., 2021).

## Conclusion

This review highlights the underlying mechanisms of arsenic toxicity on male reproduction. Mitochondria are targets for arsenic impact since they are involved in energy metabolism and ROS production. The generation of oxidative stress and inhibition of thiol-content proteins are the main molecular events involved in arsenic injuries on testicular and epididymal tissues. Overproduction of ROS creates a hostile environment in the epididymis, which enhances the lipid peroxidation in sperm membranes, thereby affecting sperm motility, viability, and fertility. Owing to the widespread exposure of humans and animals and the known toxicity of this metalloid, this review sheds light on the relevance of arsenic as a promoter of male infertility.
